# The University and Hospital Bulletin

**Published:** 1904-12

**Authors:** 


### The University and Hospital Bulletin

DEVOTED TO THE INTERESTS OF THE UNIVERSITY OF BUFFALO AND THE HOSPITALS.

EDITED BY
NELSON W. WILSON, M. D.

*Assistants:*
E. R. McGuire, M. D., Burton T. Simpson, M. D.

Under the auspices of the Faculty of the Medical Department of the University

M. D. Mann, A. M., M. D., Dean,
Charles G. Stockton, M. D.,
John Parmenter, M. D.,
Eli H. Long, M. D.,
Roswell Park, A. M., M. D., LL. D.,
Charles Cary, M. D.,
Herbert M. Hill, A. M., Ph. D.,
Herbert U. Williams, M. D.

AND

*Buffalo General Hospital* Henry Reed Hopkins, M. D.
*Hospital of the Sisters of Charity* Eugene A. Smith, M. D.
*German Hospital* Herman E. Hayd, M. D.

The American Society of Clinical Surgery held the first half of its third meeting at the General Hospital and the University of Buffalo on October 28, 1904, the meeting being in charge of Dr. Park, the local member. This society confines itself to clinical work and exhibition of cases, no papers being read at any time.

The first session of the Buffalo meeting was held at the General Hospital at 9.30 in the morning when cases of interest were presented by Dr. Park and other Buffalo surgeons. Dr. Park exhibited two very interesting gunshot wounds, illustrating the disabling powers of the modern high power rifle ball and the results when a soldier is injured at or near the centers of sensation and motion. The first case was a soldier of the 13th Regiment who was shot at San Juan, Cuba, on July 1, 1898. The ball, a Mauser, struck him about an inch and a half above the left zygoma, its course being downward and backward. It passed through the skull emerging at a point an inch below the right mastoid. The results of this wound are a partial left-sided paralysis, partial loss of sensation of the left side of the face, loss of sight of the left eye, due to corneal ulcers following the injury.

The second case was that of a soldier of the same regiment shot at the same battle, the ball passing from above downward. The bullet struck him on top of the skull midway between the forehead and the vertex. His own description of the injurv was that of a prickling sensation all over his body. There was a free flow of blood and he states that some portions of brain substance dropped into his hand when he leaned forward. Immediately afterward he fired four shots from his rifle at a Spanish sharpshooter in a tree, then became unconscious. At the present time he is totally paralysed on the left side and there is motor paralysis of the right leg.

Dr. W. C. Phelps showed a high up amputation of the thigh which made a perfect uncomplicated recovery. He also presented a case in which he had operated for removal of the Gasserian ganglion, which recovered rapidly and without complication. The patient had been entirely free from pain since the operation.

Dr. Phelps's third case was a prostatectomy in which he had done the perineal operation.

Dr. Park showed the case of carotid aneurism which was reported briefly in the Bulletin of last month. This patient has made a perfect recovery.

A very interesting series of cases was presented by Dr. Eugene A. Smith, the first of which was one in which he had operated for cancer of cecum. The operation consisted of excising the mass. His second case was one on which he had operated two and a half years ago for cancer of the pylorus. At the present time the patient is in excellent health and there has been no signs of a recurrence anywhere of cancerous growth. Dr. Smith also reported the case referred to in the Bulletin recently where he had amputated the thigh for thrombosis of the femoral artery. He also exhibited specimens of interest from his collection.

A case of thrombosis of the splenic artery was presented by Dr. Vertner Kenerson.

Dr. H. C. Rooth showed a case of fractured skull, frontal, vertex and base, with rupture of the middle meningeal artery in which he secured a perfect recovery. Dr. Rooth's second case was a gunshot wound of the left arm, the ball entering the inner surface, middle third, six weeks prior to exhibition, severing the triceps and rupturing the brachial artery. The triceps were brought together and nerve injury repaired. Four weeks after the injury motion of the hand and forearm appeared. A peculiar feature in connection with the case was that since the injury the nail of the hand of the injured arm had not grown.

Dr. Marshall Clinton presented a gallbladder case in which he had operated, a woman of 35, who had a history covering eleven years. When she came under Dr. Clinton's care she had high temperature and rapid pulse with a marked leukocytosis. The gallbladder was opened, stones removed and drainage was followed by a free flow of bile. Four days later she had a chill with colic and two small stones were discharged through the tube, having come from the cystic duct. The patient was making an uninterrupted recovery when presented.

A specimen of Meckel's diverticulum was shown by Dr. Clinton with a perforation. This case was reported in a recent issue of the Journal by Dr. Clinton. Dr. Park showed an old case of facial paralysis which he had operated. The facial nerve was divided seven years ago. It was sutured and the patient recovered with excellent result.

An interesting case of spina bifida and hydrocephalus was presented in which Dr. Park had operated on the bifida with perfect result. As yet nothing has been done regarding the hydrocephalus.

In the next case Dr. Park exhibited a thoracic-plastic operation performed thirteen years ago for empyema. The operation was widespread and there exists now considerable deformity of the chest wall, lateral left chest wall; but the patient is in such excellent condition that for the past nine years he has been doing fairly heavy work in a brickyard without serious inconvenience. In connection with this operation Dr. Park in presenting two other cases spoke of the use of brewer's yeast in pus cases which was originated at his clinic nine years ago, and had since been used with excellent results. One of the cases referred to necessitated the removal of a portion of one rib following pneumonia and a cavity containing two quarts of pus drained and packed with the yeast. In the other case pieces of five ribs were removed and a thickened pleura enclosing a pocket of pus was discovered, opened, drained and packed with yeast. Both cases recovered without complications and without delay. Two cases of malignant growth of the face, both on the left side, were presented by Dr. Park. One was a rodent ulcer, the patient being an old woman, for whom nothing could be done. The other case that of a comparatively young man who was also inoperable. He has an epithelioma which covers almost the entire side of the face and is rapidly progressing. This case originated ten months ago.

In view of the serious and in some cases fatal results following *x*-ray burns which have recently come to notice, Dr. Park's next case was of unusual interest. A young woman suffering with a tubercular wrist joint underwent *x*-ray treatment being exposed to the rays nine or ten times. A stubborn and rather widespread burn followed which ulcerated and resisted all treatment. When the case came to Dr. Park's attention he performed a skin-grafting operation and saved the hand with little deformity of the wrist. One of the most interesting of Dr. Park's cases was a prostatectomy by the suprapubic route after Freyer's method, but with modifications introduced by Dr. Park in the way of drainage which appears to lessen the chances of complication. Cathcart and Freyer both use drainage tubes, rather large in caliber, and pack these around with gauze. Dr. Park uses a much larger tube and maintains a constant siphonage with a regulating stop-cock which prevents accumulation of urine and wound secretion. The advantage of this apparatus is shown in the rapidity of the recovery. The drainage apparatus was demonstrated to the members of the society.

A surgical engine for bone cutting was shown by Dr. H. R. Gaylord who also presented a large number of nails, screws, bits of glass and other debris which he had removed from the stomach of a professional glass eater.

After the morning session Dr. Park entertained the society and a few invited guests at luncheon at the University Club. In the afternoon the society visited the University of Buffalo, the Gratwick Laboratory, and in the afternoon at the University Dr. Park delivered an illustrated lecture on "Medicine and Surgery in Classic Art." A dinner at the Buffalo Club followed and in the evening the society went to Cleveland to complete the meeting.

Those who were present at the Buffalo session were A. D. Bevan, M. L. Harris, Chicago; J. A. Blake, George E. Brewer, New York; J. C. Bloodgood, Harvey Cushing, John T. Finney, Baltimore; G. W. Crile, Cleveland; G. G. Davis, C. H. Frazier, Philadelphia; R. B. Greenough, F. B. Lund, J. G. Mumford, E. H. Nichols, J. C. Munro, Boston; W. J. Mayo, Rochester, Minn.; Roswell Park, Buffalo.

The Cleveland program, arranged by Dr. G. W. Crile, was as follows:

Breakfast at the Hollenden Hotel.

Lakeside Hospital. D. P. Allen, by invitation: clinic; C. A. Hamann, by invitation: Case of cancer of thyroid with metastasis to the skull; F. E. Bunts, by invitation: clinical case; Thomas C. Martin, by invitation: Methods of examination and treatment of certain surgical diseases of the rectum with demonstrations; W. E. Lower, by invitation: (*a*) Value of the freezing point of the blood and urine in surgery, (*b*) Demonstration of ureteral catheterisation; Hunter Robb: clinic; G. W. Crile: (*a*) Demonstration of methods of control of the blood pressure and minimising hemorrhage, (*b*) Operation for pelvic hernia, (*c*) Operation for relief of vicious circle, following gastroenterostomy.

Luncheon at Lakeside Hospital.

Western Reserve Medical Building. Demonstration of borderland surgery. C. A. Hamann, by invitation: Surgical anatomy of the gasserian ganglion; Torald Sollman, by invitation: Perfusion of the excised kidney; W. T. Howard, Jr., by invitation: Surgical endotheliomata; J. J. R. Macleod, by invitation: (*a*) Secretin injection as an aid to the diagnosis of pancreatic fistula, (*b*) The value of chemical tests for the presence of trypsin in pancreatic cysts; G. W. Crile: (*a*) Physiology of striped muscle fibers in relation to dislocations, (*b*) Hemorrhage, (*c*) Subcutaneous feeding, (*d*) Resuscitation, (*e*) Plan of teaching surgical physiology.

Dinner at the Tavern Club.

---

Dr. George E. Brewer, president of the American Society of Clinical Surgery, is attending surgeon at Roosevelt Hospital, New York. He is an alumnus of the University of Buffalo medical department, class of '81.

---

Dr. Park operated for goiter at the General Hospital, under the most improved methods, early in November, with most excellent results, following Crile's procedure in thyroidectomy. The patient was a man of 28, with left side and median enlargement of the gland. The morning of the operation his blood pressure was taken to secure the relative normal pressure for guidance during the operation. Prior to operation the patient was placed in Crile's pneumatic suit. When put on the table for operation and immediately before the incision, the head of the table was elevated, bringing the patient into what might be termed a reversed Trendelenburg position. At the same time moderate pressure was made over the body by partially filling the suit with air. This permitted the larger veins to empty themselves, preventing hemorrhage and increasing blood pressure. In this procedure the undivided attention of one assistant is necessary to control the blood pressure, which is taken at frequent intervals. If pressure falls, it is rapidly restored by increasing the amount of air in the suit. The patient's normal blood pressure was 130. At one time during the operation it fell to 120, but rose immediately to 133, with increased air pressure in the suit. Dr. Park made a semicircular incision and dissected out the entire left lobe and the isthmus with very little hemorrhage. After the patient's return to the ward, blood pressure was frequently taken, and as no marked reduction was noted, the air in the suit was gradually reduced and the suit was removed in the evening. The patient made an uninterrupted recovery and was discharged from the hospital on the seventh day after operation.

---

The following case is chiefly interesting because of its diagnostic features. For over two years the patient, a man of 56, had complained of gastric disturbance, slowly increasing in severity until at the present time he suffered so much that his only relief was secured by gastric lavage. His history was typical of pyloric obstruction with increasing difficulty until he presented himself, when there was almost total stomach stagnation, which he had been able to only partially relieve by lavage. With the noted symptoms there had been progressive and constant loss of flesh.

Under examination there could be felt a mass extending two inches beneath the free border of the ribs on the left side near the median line. The mass was hard, but not nodular, and upon air distension of the stomach became less distinct and moved to a position somewhat above the stomach. The questions to be determined, concerned the exact location of the mass whether in the stomach or not. If it was in the stomach it was not on the anterior wall, because it became less distinct on distension. If in the organ it must be posteriorly located. Then again there was a question as to whether it might not be the left lobe of the liver. This was, however, impossible to determine with any degree of satisfaction, and an exploratory incision was imperative for diagnostic purposes. The operation revealed a malignant growth of the left lobe of the liver with serious lymphatic involvement deep in the mesentery. The mass pressed upon the pylorus in such a way as to almost completely close it. The adhesions were dense and wide-spread, and the condition was plainly beyond surgical skill. A posterior gastroenterostomy was done as a palliative measure to relieve the pyloric obstruction. Recovery from this operation was uneventful.

---

THERE was brought to the General Hospital in Dr. John Parmenter's service a workman who had fallen 30 feet. He was practically in a dying condition. There was much bleeding from both ears; he was in deep coma, there was sensory and motor paralysis of both extremities, but sensation was present above the ninth dorsal vertebra, at which point there was marked angular deformity. The diagnosis was fracture of the base of the skull and fracture and dislocation of the spine at the ninth dorsal.

The patient rallied from shock quickly and for three days made rapid improvement in his mental condition, the paralysis remaining unchanged however. Operation in the beginning was not seriously considered, because of the basilar fracture, but with the improvement at the end of the third day laminectomy was done by Dr. Parmenter. He resected part of the ninth and tenth dorsal vertebras, relieving pressure and returned the patient to bed with traction at the head and both extremities. The patient's condition has markedly improved since the operation, and there is returning motion in the extremities as well as sensation.

